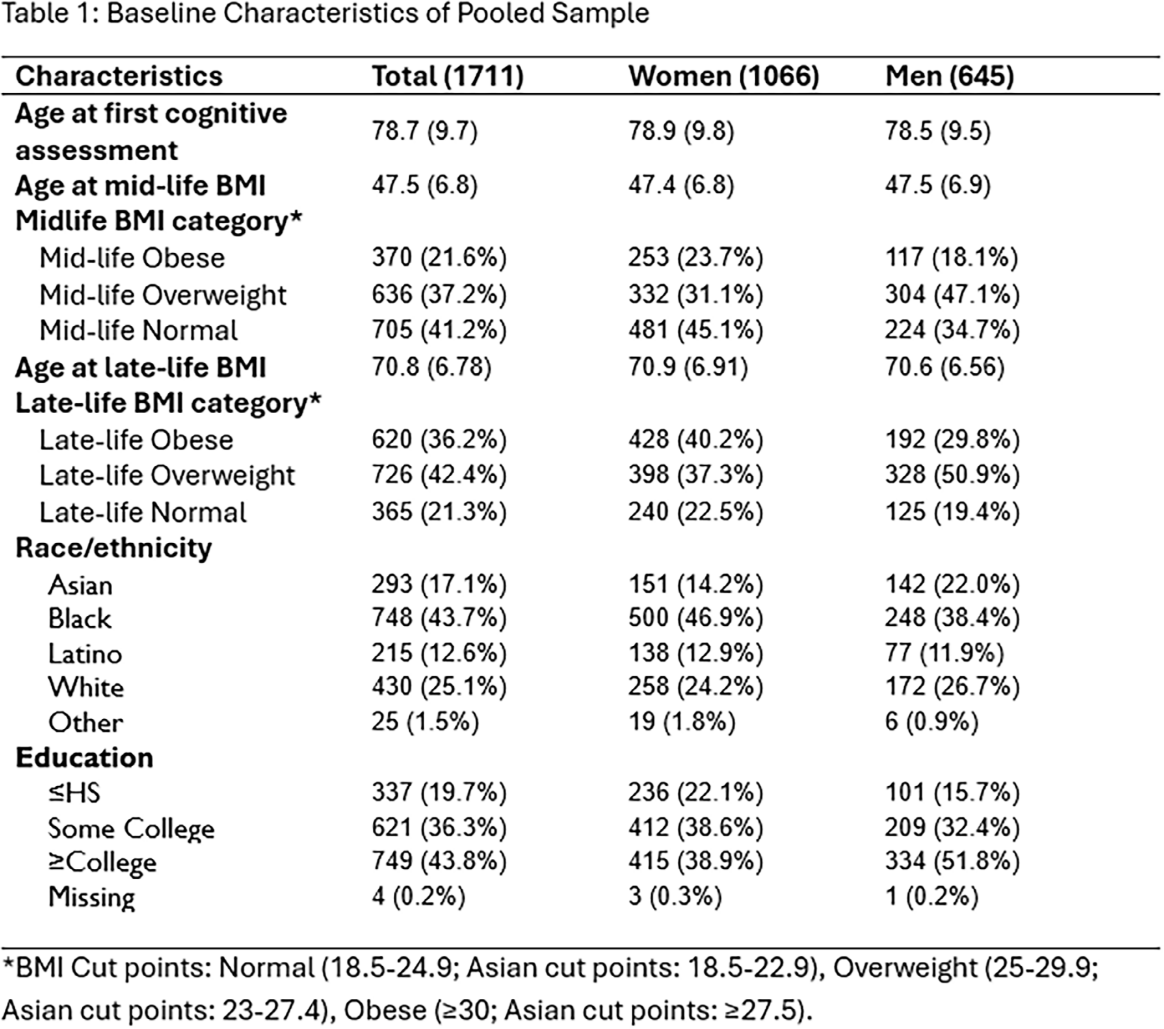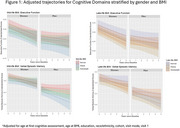# Sex/gender differences in domain‐specific cognitive decline by mid‐ and late‐life body mass index

**DOI:** 10.1002/alz70860_105096

**Published:** 2025-12-23

**Authors:** Claire C. Meunier, Paola Gilsanz, María M. M. Corrada, Kristen M. George, Michelle C Odden, Brandon E Gavett, Alexander Ivan B. Posis, Rachel A. Whitmer

**Affiliations:** ^1^ University of California, Davis, Davis, CA, USA; ^2^ Kaiser Permanente Northern California Division of Research, Pleasanton, CA, USA; ^3^ University of California, Irvine, Irvine, CA, USA; ^4^ Stanford University, Palo Alto, CA, USA; ^5^ University of California Davis, Sacramento, CA, USA

## Abstract

**Background:**

Previous research suggests sex/gender differences in the association of body mass index (BMI; kg/m^2^) with cognition, but whether the association between BMI and cognitive decline varies by gender among racially‐diverse older adults remains less understood.

**Method:**

Analyses used data from 1,711 participants in a harmonized cohort of racially/ethnically diverse older adults (LifeAfter90, KHANDLE, STAR) with BMI measured during mid‐ (ages 40‐60) and late‐life (≥65). Verbal episodic memory (VEM) and executive function (EF) were measured using z‐standardized Spanish and English Neuropsychological Assessment Scales. Mid‐ and late‐life BMI were obtained from the first available health records at each life stage and categorized into normal, overweight, and obese using race‐specific cutoffs. Separate linear mixed models explored sex/gender differences in mid‐ and late‐life BMI effects on cognitive decline using three‐way interaction (sex/gender*BMI category*time) and sex/gender‐stratified analyses. Models were adjusted for demographics, assessment‐mode (in‐person vs phone), and visit 1(Y/N) to account for practice‐effects.

**Result:**

Participants were 62% women and had a mean age of 79±9.7 at first cognitive assessment (Table 1). At mid‐life (mean BMI age=48±6.8), 22% of participants had obesity, 37% had overweight, and 41% had normal BMI. At late‐life (mean BMI age=71±6.8), 36% of participants had obesity, 42% had overweight, and 21% had normal BMI. Neither mid‐ nor late‐life BMI category were associated with EF decline, regardless of sex/gender (interaction *p* >0.10). Associations of mid‐life obese BMI, mid‐life overweight BMI, and late‐life overweight BMI on VEM decline differed by gender (*p* <0.10). Stratified analyses found that among women, those with higher mid‐life BMI had slower decline in VEM, though the difference was not significantly different than women with normal midlife BMI (overweight: (β(95%CI):0.03(‐0.03,0.08); obese:0.01(‐0.02,0.05)). Among men, those with obese BMI at mid‐life had faster decline (‐0.07(‐0.14,‐0.001)) than those with normal BMI. In late‐life, women with higher BMI (obese: ‐0.03(‐0.09,0.02), overweight:‐0.05(‐0.10,0.01)) trended towards a faster decline in VEM, whereas men with higher BMI at late‐life (obese: 0.01(‐0.05,0.08), overweight: (0.05(‐0.01,0.11)) trended towards less decline (Figure 1).

**Conclusion:**

These findings suggests that mid‐life and late‐life BMI may have differential associations with VEM in men and women. This may suggest differences in underlying mechanisms of cognitive decline between sex/gender.